# Supplementary material for: Comparison of post-COVID symptoms in patients with different severity profiles of the acute disease visited at a rehabilitation unit
Source: PLoS One. 2022 Sep 16;17(9):e0274520. doi: 10.1371/journal.pone.0274520 (PMC9481013; doi:10.1371/journal.pone.0274520)
Supplement: Attachment — Submitted filename: response to the reviewers v2.docx [file pone.0274520.s003.docx]

**RESPONSE TO THE ACADEMIC EDITOR, Peter Schwenkreis,**

1.- The first issue is the use of the term "sensitivity" with respect to the Pfeiffer questionnaire. "Sensitivity" (true positive rate) refers to the probability of a positive test, conditioned on truly being positive. Since you do not know the real proportion of patients with cognitive impairment, you must not use the term "sensitivity" or "sensitive" in this context.

**In agreement with the editor, we have changed the term "sensitivity" for the “ability”.**

2.- The second issue is the use of the term "myopathy" in your study. Apparently you use this term synonymously to muscular weakness, based on the muscle strength scale (mMRC), the short physical performance battery (SPPB), the manual dynamometry and the effort capacity with the 6-minute walk test-6MWT. However, muscular weakness as assessed by these tests is only a symptom, whereas myopathy is defined as a disease of the muscles resulting e.g. from specific inflamatory or metabolic processes. Muscular weakness is not necessarily caused by myopathy, but may also occur in deconditioned patients due to immobilization. You should therefore avoid the use of the term "myopathy" in this context, when you cannot be sure that the patients are indeed suffering from a specific muscular disease.

**Thank you for pointing this out. We agree and have now changed the term “myopathy” to “muscular weakness”.**

3.- Besides, you should include in the limitations section that "fatigue" was only assessed by patients reportings, not by standardized and validated questionnaires.

**In agreement with the editor, now we have added the following sentence in the limitations section:**

**Finally, fatigue was assessed only from the symptoms reported by the patients.**

***We thank the Editor for these helpful suggestions.***
